# Letter regarding “*ALVAC‐fIL2*, a feline interleukin‐2 immunomodulator, as a treatment for sarcoids in horses: A pilot study”

**DOI:** 10.1111/jvim.16495

**Published:** 2022-07-20

**Authors:** Mark Rishniw, Maurice White

**Affiliations:** ^1^ Department of Clinical Sciences Cornell University, College of Veterinary Medicine Ithaca New York USA; ^2^ Department of Population Medicine and Diagnostic Sciences Cornell University, College of Veterinary Medicine Ithaca New York USA

Dear Editors,

We read with interest the recent study by Saba and colleagues, “*ALVAC‐fIL2*, *a feline interleukin‐2 immunomodulator*, *as a treatment for sarcoids in horses*: *A pilot study*.”^1^ We applaud the Journal for including a category for pilot studies—such studies can provide useful information. However, the Journal's own instructions for authors define pilot studies as “*small scale studies designed to test the feasibility of future large‐scale studies and not to report on the effectiveness of an intervention*, *for which they are often underpowered*. *Research by others has identified weaknesses in the reporting and conduct of pilot studies*, *including inappropriate reporting on outcomes of interventions and not reporting on the feasibility of a larger study*.” The Journal provides a reference to an article that describes pilot studies and their utility, implementation and reporting.^2^

Two years ago, we published a critical review of pilot studies in which we demonstrated that most articles published in veterinary sciences as “pilot studies” failed to conform to the definition of a pilot study.^3^ Almost all measured effectiveness and/or safety of the intervention, and the findings were never confirmed with larger studies, but most studies were subsequently cited as evidence of effective interventions. Instead, the term appeared to be used as a synonym for “small, underpowered study” and a covert plea for leniency. We termed this “deficiency signaling.”

The study by Saba and colleagues appears to violate several of the requirements of the pilot study rules—it provides estimates of response, estimates of adverse events and concludes that the treatment is “*a safe*, *cosmetic and effective treatment for sarcoid tumors in horses*.” Furthermore, the authors provide no evidence that a larger, prospective, randomized clinical trial is being conducted, based on the methodological approaches used in the pilot study. Indeed, the study in question is a small (14 horse) case series, not a pilot study.

We would request that the Journal adhere to its own guidelines when accepting pilot studies for publication and that authors be asked to justify the use of the term “Pilot study” or “preliminary study” or “proof‐of‐concept study” (analogous terms), either in the title or text. We suspect that in most cases, authors will fail to satisfy the requirements these terms demand.

Sincerely,
